# Design, implementation, and evaluation of a knowledge translation intervention to increase organ donation after cardiocirculatory death in Canada: a study protocol

**DOI:** 10.1186/1748-5908-9-80

**Published:** 2014-06-20

**Authors:** Janet E Squires, Jeremy M Grimshaw, Monica Taljaard, Stefanie Linklater, Michaël Chassé, Sam D Shemie, Gregory A Knoll

**Affiliations:** 1 Ottawa Hospital Research Institute, Clinical Epidemiology Program, 501 Smyth Road, Room 1282, Box 711, Ottawa, ON K1H 8 L6, Canada; 2 School of Nursing, University of Ottawa, Ottawa, Canada; 3 Department of Medicine, The Ottawa Hospital/University of Ottawa, Ottawa, Canada; 4 Department of Epidemiology and Community Medicine, University of Ottawa, Ottawa, Canada; 5 Division of Critical Care, Montreal Children’s Hospital, McGill University Health Centre, Montreal, Canada; 6 Faculty of Arts, University of Ottawa, Ottawa, Canada; 7 Department of Pediatrics, McGill University, Montreal, Canada

## Abstract

**Background:**

A shortage of transplantable organs is a global problem. There are two types of organ donation: living and deceased. Deceased organ donation can occur following neurological determination of death (NDD) or cardiocirculatory death. Donation after cardiocirculatory death (DCD) accounts for the largest increments in deceased organ donation worldwide. Variations in the use of DCD exist, however, within Canada and worldwide. Reasons for these discrepancies are largely unknown. The purpose of this study is to develop, implement, and evaluate a theory-based knowledge translation intervention to provide practical guidance about how to increase the numbers of DCD organ donors without reducing the numbers of standard NDD donors.

**Methods:**

We will use a mixed method three-step approach. In step one, we will conduct semi-structured interviews, informed by the Theoretical Domains Framework, to identify and describe stakeholders’ beliefs and attitudes about DCD and their perceptions of the multi-level factors that influence DCD. We will identify: determinants of the evidence-practice gap; specific behavioural changes and/or process changes needed to increase DCD; specific group(s) of clinicians or organizations (*e.g.*, provincial donor organizations) in need of behaviour change; and specific targets for interventions. In step two, using the principles of intervention mapping, we will develop a theory-based knowledge translation intervention that encompasses behavior change techniques to overcome the identified barriers and enhance the enablers to DCD. In step three, we will roll out the intervention in hospitals across the 10 Canadian provinces and evaluate its effectiveness using a multiple interrupted time series design.

**Discussion:**

We will adopt a behavioural approach to define and test novel, theory-based, and ethically-acceptable knowledge translation strategies to increase the numbers of available DCD organ donors in Canada. If successful, this study will ultimately lead to more transplantations, reducing patient morbidity and mortality at a population-level.

## Background

### The Canadian National Transplant Research Program (CNTRP)

This protocol is part of the Canadian National Transplant Research Program (CNTRP), an integrated national coalition of investigators formed to address challenges with solid organ transplantation and hematopoietic stem cell transplantation. The vision of CNTRP is to transform the overall effectiveness of transplantation in Canada by increasing transplant numbers and improving long-term graft and patient survival through collaborative multi-disciplinary research. The CNTRP unites teams of basic and clinical scientists in organ donation, hematopoietic stem cell transplantation, and liver, heart, lung, pancreas, and kidney transplantation, as well as knowledge translation, health economics, legal and ethics researchers, policy experts, and knowledge users. The CNTRP projects span the major organs and cell types transplanted, with an integrated national perspective and participation across nine Canadian provinces. This dynamic research coalition integrates six projects and two comprehensive supporting cores:

Project one: *Ex vivo* organ transplant protection and repair;

Project two: Increasing solid organ and hematopoietic cell donation in Canada;

Project three: Favoring engraftment and preventing rejection/graft-*vs*-host disease through targeted disruption of danger and death signals: from cells to patients;

Project four: Translating strategies for immunomodulation and transplantation tolerance;

Project five: Viral pathogenesis in transplantation: prediction, discovery and optimization of risk;

Project six: Personalizing immunosuppression to improve age-related transplant outcomes;

Core one: Ethical, economic, legal and social issues in transplantation;

Core two: Platforms supporting transplant correlative studies, database registries, training and interventional clinical trials.

The CNTRP provides a unique opportunity to study important issues in donation with immediate clinical impact with respect to increasing, in a low-risk and ethical manner, the number of organs available for transplant. The protocol described in this manuscript is part of project two (increasing solid organ and hematopoietic cell donation in Canada) and details a study that aims to develop and test a theory-based, multi-faceted knowledge translation intervention implemented at the hospital level to increase the number of donation after cardiocirculatory death organ donors for transplantation across Canada.

### The problem

A shortage of transplantable organs is a global problem. Despite Canada’s envied healthcare system and economic status amongst developed nations, we perform very poorly in organ donation. There are two types of organ donation: live donation when a living person donates an organ or part of an organ to another individual and deceased donation when an individual becomes a donor following their death. Deceased donation can occur after neurological determination of death (NDD) or cardiocirculatory (or cardiac) death (DCD). DCD was not used routinely in Canada until 2006. In 2010, the deceased donor rate in Canada was 13.6 per million population (PMP) [1] compared to 32 PMP in Spain and 25 PMP in the United States, with no improvement in the past decade (2001, 13.1 PMP) [1]. While Canada has had 465 to 485 deceased donors yearly over the past five years [1], 4,500 Canadians remain active on transplant waiting lists [2,3].

Use of DCD donors is frequent worldwide and accounts for the largest increments in deceased organ donation. Variations in the use of DCD continue, however, in jurisdictions within Canada and worldwide [2,4]. Since 2006, DCD has grown in some Canadian jurisdictions but has remained stagnant or absent in others. In 2011, DCD accounted for 20% of all deceased organ donors in Ontario (3.1 DCD donors PMP), 9.5% in Quebec (1.6 PMP), 8.7% in BC (1.5 PMP), 8% in Alberta (0.8 PMP), 8% in Nova Scotia (2.1 PMP), and 0% in Saskatchewan, Manitoba, New Brunswick, Prince Edward Island, and Newfoundland [2,5]. Compared to other jurisdictions, even Ontario’s ‘high’ DCD rate is relatively low. The DCD rate in the UK is 5.2 PMP with some regions reporting rates up to 12.3PMP [6]. Canada would see a dramatic increase in the number of available organ donations if DCD donation rates were to increase to international standards. Reasons for the wide international variations in DCD uptake remain unknown.

The number of overall transplant candidates in Canada has grown each year, but the number of deceased donors has not kept pace, leading to more wait-list deaths. In 2010, 16% of kidney-pancreas, 19% of lung, 22% of liver, and 24% of heart transplant candidates died on a Canadian wait-list before receiving a transplant [2]. There are even wait-list deaths amongst patients with end-stage renal disease who can be maintained on dialysis: 101 deaths in 2010 alone [2]. This growing imbalance between supply and demand for organs means that Canadians needing a transplant face a 30% to 40% lifetime probability of never receiving one [7]. The World Health Organization has charged member nations to begin to address this serious medical health issue by, among other objectives, improving their national systems for both living and deceased donation [8,9].

### Barriers and enablers to donation after cardiocirculatory death (DCD)

Reasons for low DCD rates are understudied and as a result, poorly understood. Perceived barriers to DCD, suggested in a limited number of studies, include: varying clinician attitude, time and logistical constraints, inability to predict time of death to optimize DCD, unknown incidence of auto-resuscitation, and ethical/legal concerns about violation of the ‘dead donor rule’ which states that organ retrieval itself cannot cause death [5,10-12]. Recently, a more comprehensive study was undertaken across the United States to identify different clinicians’ views of the barriers and enablers to their ‘acceptance’ of DCD [13]. This study found several key barriers to DCD acceptance, including: lack of knowledge about DCD, psychological barriers for DCD versus NDD, concerns about recognition of death, saving versus ‘killing’ patients, trust in the organ procurement organization, moving from saving patients to being a donation advocate, and concerns with the DCD process generally. Enablers to the acceptance of DCD included: education initiatives, well-trained individuals whom request the organ donation with families, a cultural shift, a consistent DCD protocol separating care from recovery, process monitoring, and a strong sense of teamwork [13]. No similar Canadian studies on clinician attitudes and beliefs to DCD were located.

In summary, the barriers and enablers to DCD have not been well explored. Further, no studies have specifically investigated DCD from a behavioural theory approach which encompasses both barrier and enabler assessment of a broad range of the possible multifactorial determinants of the behaviour. Therefore, the aims of this study are, first, to use behavioural perspectives to help identify the barriers and enablers to DCD as perceived by healthcare professionals, and then to develop, implement, and evaluate the effectiveness of a theory-based knowledge translation intervention at the hospital level to increase the numbers of DCD organ donors (without reducing the number of NDD organ donors) for transplantation in Canada.

### Guiding frameworks

We have adopted two overarching frameworks to guide our study. First, we will use the Knowledge-to-Action Framework that highlights the central processes related to knowledge creation, distillation and use [14]. It is comprised of a Knowledge Creation funnel and an Action Cycle. The Action Cycle is based on planned action theories that focus on deliberate engineering change in healthcare systems and groups. Processes needed to successfully implement knowledge into clinical practice, namely: problem clarification; identifying the determinants of the knowledge-action gap; selecting, tailoring, implementing, and evaluating knowledge translation interventions; and determining strategies for ensuring sustained knowledge use are included. Second, we will use the UK Medical Research Council Complex Interventions Framework [15,16], which provides an iterative phased approach to the development and evaluation of complex (such as knowledge translation) interventions. This framework states that the development and evaluation of complex interventions should follow a sequential approach:

Phase zero: problem and contextual assessment, and development of the theoretical basis for an intervention;

Phase one: definition of components of the intervention (using modeling or simulated techniques and qualitative methods);

Phase two: exploratory studies to further develop the intervention and plan a definitive evaluative study (using a variety of methods);

Phase three: definitive evaluative studies (using quantitative evaluative methods, predominantly randomized designs); and,

Phase four: studies evaluating the sustainability of complex interventions.

The study described in this protocol addresses Phases zero to three of the Medical Research Council Complex Interventions Framework and the Action Cycle of the Knowledge-to-Action Framework in order to develop, implement, and evaluate a knowledge translation intervention to increase the number of DCD donors across Canada.

## Methods

### Design and objectives

This is a five-year multi-phased study that will use state of the art approaches from knowledge translation science and health psychology to systematically develop a theory-based knowledge translation intervention to change institutional, team, and clinician behavior in order to increase the number of DCD donors in Canada. This population-level evaluation study will use a multiple interrupted time series design to assess the effectiveness of the intervention in the 10 Canadian provinces: British Columbia, Alberta, Saskatchewan, Manitoba, Ontario, Quebec, New Brunswick, Nova Scotia, Prince Edward Island, and Newfoundland.

Our specific objectives are to:

1. Understand the process of, and identify the determinants to, DCD as perceived by healthcare professionals (organ donor coordinators, intensive care nurses, intensive care physicians (intensivists)) across Canada *(*Medical Research Council Complex Interventions Framework- Phase zero, Knowledge-to-Action- assessing determinants of knowledge translation*)*

2. Develop a theory-based hospital-level knowledge translation intervention to increase the number of DCD organ donors in Canada that is based on the identified determinants (Medical Research Council Complex Interventions Framework—Phases one and two, Knowledge-to-Action - selecting, tailoring knowledge translation interventions)

3. Conduct a national implementation and evaluation for effectiveness of the knowledge translation intervention using a multiple interrupted time-series design (Medical Research Council Complex Interventions Framework—Phase three, Knowledge-to-Action—implementing and evaluating knowledge translation interventions)

### Objective one: understand the process of, and identify the determinants to, DCD as perceived by healthcare professionals across Canada

The purpose of this initial step is to identify and describe key informants’ beliefs and attitudes about DCD and their perceptions of the multi-level factors that influence this behaviour. This will allow us to identify and understand: the complex process of DCD, including decisions around withdrawing life-sustaining treatment and subsequent organ donation or not; the determinants of the evidence-practice gap (*i.e.*, why DCD is not used consistently across Canada); what specific behaviors and/or processes need changing in order to increase DCD; which specific group or groups of clinicians behavior needs changing (*e.g.* donor coordinators, critical care nurses, critical care physicians); and the specific targets that need to be addressed in a knowledge translation intervention. This necessary preliminary work will generate a thorough understanding of healthcare professionals’ perceptions of the multi-level determinants of the behaviours required to improve DCD in Canada, which is critical to the development of successful knowledge translation interventions.

### Data collection

Semi-structured interviews informed by an interview guide will be conducted with key informants (intensivists, intensive care unit nurses, and organ donor coordinators) across all regions of Canada to gather data on the barriers and enablers to DCD. We chose this approach for three reasons. First, it allows participants to respond freely, to illustrate concepts, and to present individual perspectives that the interviewer can probe further [17]. Second, a semi-structured interview guide will increase the likelihood that busy participants cover the topics of interest in an efficient manner [18]. Third, such a guide facilitates flexibility, such that an interviewer may explore in greater depth issues that may arise in the interview that are not addressed by the interview guide [19,20]. Development of the interview guide will be informed by the Theoretical Domains Framework (TDF), a behavior change framework comprised of 14 ‘theoretical domains’ derived from 128 constructs from 33 health and social psychology theories that explain health-related behavior change [21,22]. This framework has been used to identify the determinants of a wide range of professional behaviors [18,23-29]. The theoretical domains offer wide coverage of the potential multi-level determinants of health-related behaviors and guide the use of broad prompts that enable interviewees to consider a wide range of possibilities without asking leading questions [18]. Definitions of the 14 theoretical domains are in Table 1. The TDF-informed interview guide will be used in this study to probe key informants about reasons they do or do not accept/use DCD consistently in their clinical practice. This will allow us to identify key beliefs from different behavioural domains that could be targeted by knowledge translation interventions to increase DCD. The interview guide was pilot-tested with three intensivists and two intensive care unit nurses. The pilot interviews were used to shorten the interview guide, as well as revise the wording of some of the questions to ensure they were clear, easy to comprehend, and applicable to the three targeted key informant groups (organ donor coordinators (often nurses), intensive care unit nurses, and intensivists). The final (revised) interview guide is in Additional file 1.

**Table 1:** The 14 theoretical domains of the theoretical domains framework

**Theoretical domain**	**Definition**[21]
Knowledge	An awareness of the existence of something
Skills	An ability or proficiency acquired through practice
Social/professional role and identity	A coherent set of behaviours and displayed personal qualities of an individual in a social or work setting
Beliefs about capabilities	Acceptance of the truth, reality, or validity about an ability, talent, or facility that a person can put to constructive use
Optimism	The confidence that things will happen for the best or that desired goals will be attained
Beliefs about consequences	Acceptance of the truth, reality, or validity about outcomes of a behaviour in a given situation
Reinforcement	Increasing the probability of a response by arranging a dependent relationship, or contingency, between the response and a given stimulus
Intentions	A conscious decision to perform a behaviour or a resolve to act in a certain way
Goals	Mental representations of outcomes or end states that an individual wants to achieve
Memory, attention and decision processes	The ability to retain information, focus selectively on aspects of the environment and choose between two or more alternatives
Environmental context and resources	Any circumstance of a person’s situation or environment that discourages or encourages the development of skills and abilities, independence, social competence, and adaptive behaviour
Social influences	Those interpersonal processes that can cause individuals to change their thoughts, feelings, or behaviours
Emotion	A complex reaction pattern, involving experiential, behavioural, and physiological elements, by which the individual attempts to deal with a personally significant matter or event
Behavioural regulation	Anything aimed at managing or changing objectively observed or measured actions

The study research assistant (SL), who is experienced in conducting structured interviews using the TDF [18], will conduct the interviews. Interviews will be conducted by telephone, digitally recorded (with participants’ consent) and are expected to last approximately 20 to 30 minutes. Before beginning each interview, informed consent will be obtained from each key informant. Key informants will be given $30 in appreciation of their time.

### Sampling procedures

We expect to interview approximately 60 key informants from across Canada: 40 clinicians (intensivists and intensive care unit nurses) and 20 organ donor coordinators working for Canadian Organ Procurement Organizations (Table 2). In qualitative research, there are no hard and fast rules about sample sizes. Determining adequate sample size is ultimately a matter of judgment and experience in evaluating the quality of the information collected against the uses to which it will be put, the particular research method and purposeful sampling strategy employed, and the research products intended [18,30]. We will use the concept of data saturation and conduct interviews within each of the three key informant groups identified above until no new information is being offered (likely at around 20 interviews per group [20,31]). We will consider saturation to have occurred when no new themes emerge on three successive interviews (for each key informant group) [32].

**Table 2:** Proposed key informant sample distribution

**Key informant group**	**Minimum number of interviews to be conducted**				
	**Atlantic Canada**^**1**^	**Quebec**	**Ontario**	**Western Canada**^**2**^	**Total interviews**
Intensivists	5	5	5	5	20
Intensive care unit nurses	5	5	5	5	20
Organ donor coordinators	5	5	5	5	20
Total interviews	15	15	15	15	60

^1^Atlantic Canada = New Brunswick, Nova Scotia, Prince Edward Island, Newfoundland.

^2^Western Canada = British Columbia, Alberta, Saskatchewan, Manitoba.

Key informants will be selected using a purposive sampling strategy augmented with snowball sampling. Sampling will focus on obtaining information-rich cases, while ensuring that each Canadian province and key informant group is represented within the sample. Recruitment will commence with the intensivist group. A master list of intensivists for all Canadian provinces was generated through discussion and communication with intensivists within the CNTRP. We will purposefully recruit intensivists that have strong opinions both for and against the use of DCD to ensure we elicit a range of both barriers and enablers to the behavior. Initial contact will be by email, during which time the study purpose and data collection process will be explained and participation requested. The remaining two key informant groups (intensive care unit nurses and organ donor coordinators), will be recruited using snowball sampling. We will contact the nurse managers of the healthcare facilities that the interviewed intensivists come from, who will be asked to suggest two to three eligible nurses and an organ donor coordinator that might be interested in participating in the study (snowball technique). The nurse manager will be provided with a study information sheet to distribute to these potential key informants that contains the contact information of the study research assistant (SL) and the study leads for this phase of the study (GAK, JES). Nurses and organ donor coordinators interested in participating will be asked to contact the study research assistant for additional information or to schedule an interview.

### Analysis plan

The interviews will yield a large quantity of data. To monitor the progress of the interviews and permit follow-up of issues that may emerge from the data, interviewing, transcription, and analysis will occur concurrently in this phase of the study. The digital recordings will be transcribed verbatim and verified by the interviewer prior to analysis. Nvivo [33] software will be used to manage the data. Analysis will occur in three steps [23,24].

### Step one: coding

Two team members will independently code the transcripts into the 14 TDF domains. They will meet after every five interviews to review their coding and seek consensus. Coder reliability will be assessed as the number of agreements/(total number of agreements + disagreements) [34]. Level of agreement should meet or exceed 70% [34]. If agreement is not achieved, the text will be allocated to all domains identified by both coders.

### Step two: generation of specific beliefs

A ‘specific belief’ is a collection of participant responses with a similar underlying theme that suggests a problem and/or influence on the target behaviour [23,24,35]. Specific beliefs will be generated by TDF domain for each key informant group by one team member and double-checked for accuracy by a second team member.

### Step three: identification of relevant domains

TDF domains that meet the following criteria will be judged as ‘relevant’ for the intervention: 1) relatively high frequency of specific beliefs; 2) presence of conflicting beliefs; and, 3) evidence of strong beliefs that may impact on the behavior [18,23,24,35]. The study research assistant (SL) and the knowledge translation researchers on the team (JES, JMG) will be responsible for criteria one and two, while the clinician researchers on the team (GAK, MC, SDS) will have primary responsibility for criterion three.

### Objective two: development of a theory-based hospital-level knowledge translation intervention to increase the number of DCD organ donors in Canada that is based on the identified determinants

Using the data obtained in objective one, we will develop a theory-based knowledge translation intervention that encompasses behavior change techniques to overcome the identified barriers and enhance the enablers to DCD. We will use the principles of intervention mapping, a formal systematic process for building interventions based upon determinants to behaviour change [36], to design the intervention. A three step procedure will be followed. First, the team will meet to prioritize the relevant TDF domains. The ‘relevant’ domains (from the analysis in objective one) will be presented to the team. After group discussion, team members will be asked to prioritize the domains on a nine-point scale [37,38]*.* We will regard domains with an overall rating of seven to nine (without disagreement) as ‘high priority’ for our intervention [38]. Second, the knowledge translation researchers on the team (JES, JMG) along with the study research assistant (SL) will map behaviour change techniques onto the belief statements in the ‘high priority’ domains from step one above using the Michie Behavior Change Matrix [39], which comprises a list of 53 effective behaviour change techniques. Third, a meeting of the research team along with provincial organ donor coordinators and intensivists will occur to finalize the content of the KT intervention and determine possible modes of delivery of the intervention. The intervention techniques chosen and delivery will be based on: empirical evidence and expert consensus of the effectiveness of the behavior change techniques; what is likely to be feasible in our context; and what is locally relevant and acceptable. There are many potential delivery modes in the clinical setting for most behavior change techniques, including facilitated workshops, educational meetings, educational outreach (or academic detailing), local opinion leaders, reminder systems, and audit and feedback [40]. As an example, we might use a facilitated workshop led by a respected clinician with extensive DCD experience. Follow-up from the workshop may include personal auditing and feedback of performance with respect to DCD. These are just examples; the content and delivery of the intervention will be determined jointly by the research team and provincial organ donor coordinators, and in consideration of the barriers and enablers data and the mapping process to be undertaken.

### Objective three: national implementation and evaluation for effectiveness of the knowledge translation intervention using a multiple interrupted time-series design

The final phase of our study involves rolling out the knowledge translation intervention developed for objective two in hospitals across the 10 Canadian provinces and evaluating it using a multiple interrupted time series (ITS) design. ITS is a powerful design to evaluate the effect of an experimental intervention or policy change that is introduced at a specific point in time, when repeated measures of an outcome of interest are available before and after the introduction of the intervention, and when randomization is not feasible. Segmented regression analysis is used to control for secular trends by estimating changes in intercepts and slopes from before to after the introduction of the intervention. Both an immediate impact (change in intercept) and gradual changes over time (change in slope) can be detected [41]. This design is immune against common threats to internal validity of observational study designs, including maturation and regression to the mean.

### Outcome measures

The primary outcome measure for evaluation of the knowledge translation intervention will be total donation rate (defined as the total number of organ donors, NDD plus DCD combined) per million population (PMP) per six months. Although a change in the DCD rate might seem to be a more appropriate outcome measure, we chose the total donation rate for two reasons. First, there is a concern that increased use of DCD might lead to fewer NDD donors [42]. If our intervention was successful at increasing DCD donors but led to a reduction in NDD donors, this would not be considered a positive intervention that should be adopted. If this occurred, use of the DCD rate alone as the primary outcome could potentially mislead stakeholders that our intervention should be implemented. By using the total donation rate, we will judge our intervention to be successful only if total donors increase. We hypothesize that there will be an increase in the total donation rate that is driven by an increase in the DCD rate with a neutral or even positive effect on the NDD donor rate as has been recently seen in the province of Ontario [43]. Second, since routine use of DCD only resumed in 2006 in Canada, observed DCD counts were all zero until 2005 and too small thereafter (<5 per million) to allow stable analysis of six-month intervals as planned (only seven pre- and three post-intervention time points for analysis). In contrast, the number of time points available for NDD and total donations is at least 30 (24 pre-, six post-intervention). So from a statistical perspective as well, total donation rate is the preferred primary outcome. Secondary outcome measures will be DCD rate (PPM per year) and (NDD) rate (PPM per six months).

### Analysis plan

The proposed method of analysis is a segmented linear regression autoregressive error model of the observed aggregate outcomes in each of four regions that comprise the 10 Canadian provinces: Atlantic Canada (New Brunswick, Nova Scotia, Prince Edward Island, and Newfoundland), Ontario, Quebec, and Western Canada (British Columbia, Alberta, Saskatchewan, and Manitoba). This approach is commonly used in health services research because it can tolerate fewer time points than autoregressive integrated moving average (ARIMA) models and is amenable to intuitive graphical presentation [41]. The first step of the analysis will be a graphical inspection of donation rates over time, at six-month intervals, in each of the study regions. The full model will be fitted by specifying a pre-intervention intercept and slope, and two parameters that represent the change in intercept and change in slope after introduction of the intervention. Maximum likelihood estimation will be used as it is considered one of the most appropriate methods of estimation for small samples with autocorrelated errors [44]. The analysis will adjust both parameter and variance estimates for autocorrelation. No adjustment for seasonal trends is anticipated.

The significance of the knowledge translation intervention will be evaluated using a likelihood ratio test of the change parameters. The null hypothesis is that both intercept and slope changes are zero; the alternative hypothesis is that at least one of intercept or slope change is non-zero. The immediate and gradual effect of the knowledge translation intervention will be estimated using confidence intervals for both absolute and relative change in the intercept and slope, using methods developed by Zhang *et al.*[45]. The overall effect, combining intercept and trend changes, will be estimated using the absolute and relative difference in the predicted post-intervention outcome versus the predicted outcome from the secular trend (counterfactual estimate) together with confidence intervals, using the methods developed by Zhang *et al.*[45]. As recommended, we will report 95% confidence intervals using the 99% critical value to account for the fact that time series with a small number of time points and large autocorrelation may produce artificially narrow confidence intervals.

To assess the fit of the final model, we will examine residual plots around the predicted regression lines. The residual plots will be used to check whether the linear assumption for the segmented regression model is satisfied. If the linear assumption is not satisfied, either log-transformation of the outcome or Poisson regression with population size as an offset term will be carried out.

Due to the limitations of our sample size (a minimum of 30 time points) we will conduct sensitivity analyses using methods recently developed for short interrupted time series. First, we will use the double-bootstrap approach developed by McKnight *et al.*[46] who demonstrated that empirical coverage and type I error rates are close to the nominal levels for as few as 20 observation times. Second, we will use the restricted maximum likelihood (REML) approach together with Kenward-Roger degrees of freedom investigated by Forbes *et al.*[47]. They showed that type I error rates of this approach are close to nominal size with as few as 30 observation times. Finally, for the secondary analysis of DCD rates, we anticipate only three available post-intervention observation points (at annual intervals) which is insufficient to reliably estimate a parametric trend after the intervention. Following Bloom [48], we will specify post-intervention time for DCD as a categorical variable (*i.e.*, post-intervention change will be estimated as deviations from the pre-intervention trend in post-intervention years one, two, and three).

### Power calculations

Power calculations were carried out using the simulation approach developed by Zhang *et al.*[49]. The assumptions for the power calculations were as follows: mean square error (MSE), baseline intercept, and trend equal to the observed pre-intervention values in each region; change in intercept = 0; alpha = 5%; 24 data points before and six after the intervention (N = 30 data points in total). Zhang and colleagues [49] recommend a plausible range of r = 0.1 to r = 0.5 for the autocorrelation parameter [AR(1)]. To assess sensitivity with respect to the strength of autocorrelation, we conducted the power calculations for r = 0.4 as well as very strong correlation of r = 0.8 (Table 3). A summary of the required effect sizes **Δ** (change in slope) to yield 80% power for the likelihood ratio test of the intercept and slope changes in each region are presented in Table 3 as both absolute and relative changes. With at least 30 time points and assuming moderate autocorrelation, we will have 80% power (α = 0.05) to detect annual% changes in total donations of 8.5% in the West, 5.6% in Quebec, 7.8% in Ontario, and 14.0% in the Atlantic Provinces.

**Table 3:** **Power calculation**^**1 **^**for ITS study**

	**Δ**^**3**^**donation rate (per million population per six months)**		**Δ donation count (per year based on 2011 population size)**^**3**^		**%Δ donation count (per year relative to 2011)**^**3**^	
AR(1)^2^	r = 0.4	r = 0.8	r = 0.4	r = 0.8	r = 0.4	r = 0.8
West	0.47	0.70	10.0	14.9	8.5%	12.6%
Quebec	0.48	0.73	7.7	11.7	5.6%	8.5%
Ontario	0.64	0.95	17.1	25.4	7.8%	11.5%
Atlantic	1.25	1.87	5.9	8.8	14.0%	21.0%

^1^The assumptions for the power calculations were as follows: (a) mean square error (MSE), baseline intercept, and trend equal to the observed pre-intervention values in each region; (b) change in intercept = 0, (c) alpha = 5%; (d) 24 data points before and six after the intervention (N = 30 data points in total).

^2^AR(1) = autocorrelation parameter.

^3^Δ = required effect sizes (change in slope).

### Timeline

Objective one (identification and description of: healthcare professional beliefs and attitudes to DCD, specific behaviours and groups requiring changing to increase DCD, and barriers and enablers to DCD) will be completed in year one of the study. Objective two (intervention design) and the intervention implementation will occur in year two of the study. Intervention effectiveness will be assessed in years three through five.

### Ethics

Ethics approval for this study was obtained from the Ottawa Hospital Research Ethics Board (Protocol # 20130635-01H).

## Discussion

The use of DCD in Canada is widely variable and less than that observed in other jurisdictions for reasons that remain unclear. This paper describes a study where we will assess the reasons for these discrepancies nationally to design, implement, and evaluate a theory-based knowledge translation intervention that will aim to increase the number of DCD donors in Canada. This study comprises a detailed and theory-based Canada-wide assessment of stakeholder (clinicians and donor coordinators) beliefs and attitudes to DCD, followed by intervention mapping to design the intervention, and an interrupted times series assessment over three years to evaluate the effectiveness of the developed intervention. This study holds the potential of sharply increasing the number of DCD donors and even perhaps NDD donors available in Canada, which will vastly impact patient health at the population (national) level.

This is the first study to use a behavioural theory approach to define and test novel, theory-based, and ethically-acceptable knowledge translation strategies to increase the numbers of available organ donors. Adopting a behavioural approach will lead to a better understanding of the rationale for specific behaviours related to DCD, resulting in a comprehensive framework on which to develop interventions that may be more successful in increasing the DCD organ donor pool. This study corresponds to Phases zero through three of the UK’s Medical Research Council framework for developing complex interventions [15,16]. The framework developers assert that a successful intervention is more likely when these phases are conducted as part of a larger iterative study (as proposed in this protocol), rather than as sequential studies [16]. They also argue that insights gained during this process can make valuable contributions not only to effecting change in the target population but also to the science of knowledge translation [16]. As far as we know, this will be the first Canadian assessment of the barriers and enablers to DCD. We will also have designed, rolled out across a country and robustly evaluated the effectiveness of a theory-based knowledge translation intervention that addresses these barriers/enablers to improve the number of DCD donors. The results of this study will inform future policy and practice regarding DCD in Canada.

## Abbreviations

CNTRP: Canadian National Transplant Research Program; DCD: Donation after cardiocirculatory death; ITS: Interrupted time series; NDD: Neurological determination of death; PMP: Per million population; TDF: Theoretical domains framework.

## Competing interests

The authors declare that they have no competing interests.

## Authors’ contributions

GAK, JES, JMG, MT, MC, and SDS participated in conceiving this study and securing its funding. JES drafted the manuscript; MT drafted the ITS analysis plan. All authors provided input into the protocol, critical feedback on the manuscript, and approved the final manuscript.

## Supplementary Material

Additional file 1 Increasing Organ Donation After Cardiocirculatory Death in Canada. Click here for file
