# Supplementary material for: Design, implementation, and evaluation of a knowledge translation intervention to increase organ donation after cardiocirculatory death in Canada: a study protocol
Source: Implement Sci. 2014 Jun 20;9:80. doi: 10.1186/1748-5908-9-80 (PMC4082291; doi:10.1186/1748-5908-9-80)
Supplement: Additional file 1 — Increasing Organ Donation After Cardiocirculatory Death in Canada. [file 1748-5908-9-80-S1.pdf]

Increasing Organ Donation After Cardiocirculatory Death in Canada

Interview Guide

Explanation

Thank you for agreeing to speak with me today about organ donation after cardiocirculatory death, hereafter referred to as DCD. There may appear to be overlap between questions but each question is worded to obtain specific information and so you may find that answers are repeated. It is important to note that there are no right or wrong answers to the questions and that no one will know what your specific answers were. The interview should take approximately 30 minutes and will be audio-recorded to ensure that all key points are accurately documented. Any identifying information (for example the names of individuals) that you use in the course of our discussion will be removed from the interview transcripts. If you wish to end the interview before I have asked all of the questions or if you wish to withdraw from the study you are free to do so.

I'd like to start with some basic background questions:

- Male or Female (to keep track of, won't be asked)
- How long have you been a physician/nurse/organ donor coordinator?
    i. For physicians, when did you finish medical school?
- For physicians, where did you do your ICU residency (hospital)?
- Are you comfortable providing your age?
- What hospital do you currently work at?
- How often does a potential organ donation scenario occur at your location? (any organ donation)
- Have you ever been actively involved in a DCD case?
    i. What was your role?
- Do you think that DCD should be an option? (Goal)

For the rest of the interview I have some more specific questions about DCD. When answering these, please keep in mind that we are more interested in your views on DCD than other types of organ donation.

Part 1

- Are you aware of any published practices or procedures regarding DCD? (national, provincial, institutional) (*Knowledge*)
    i. Where did you learn about them?
- In your career, how many DCD cases have you seen? (*Skills*)
- Take me through the DCD process and your role in the process, starting with how to decide to withdraw life sustaining therapies (WLST) (prompts: end of life care, deciding to offer DCD, contacting organ donor coordinator) (*Skills*)
- What skills and experience are required to use DCD properly? (*Skills*)

Part 2

- Does your institution support DCD? (*Soc. Prof. Role*)
- Is considering DCD a standard part of your practice? (*Soc. Prof. Role*)
- Is considering DCD an automatic part of your practice, or do you need to be reminded to do it? (*Memory*)
- Is there anything in your profession (ie. MD, ICU Nurse, ODC) that influences your decision to consider DCD or not? (ie. Special training, a protocol, other technologies) (*Soc. Prof. Role*)
- What aspects of your work environment influence whether or not you consider DCD? (prompts: ie. Material resources, unit culture, team, events) (*Environment*)

Part 3

- How easy or difficult is it to begin or follow the DCD process? (prompt: WLST, following DCD criteria) (*Belief about Capabilities*)
    i. What makes it easy, what makes it difficult?
- What would make it easier for you to use DCD? (prompts: guidelines, institutional support) (*Environment*)
- How confident are you in your ability to: (*Belief about Capabilities*)
    i. Carry out your role in the DCD process?
    ii. Properly decide if a patient is viable for DCD?
        – Is properly deciding which patients are viable for DCD easy or difficult? (*Memory*)
            a. If difficult, why?
            b. If easy, why?

Beliefs about consequences

- What are the benefits to DCD? (prompts: patients, donors, yourself)
- What are the negative aspects to DCD? (prompts: patients, donors, yourself)

Intentions

- Do you think about DCD when a patient suitable for the process is dying?
- Do you intend to use DCD when a patient is viable for the process?

Social influences

- Do the expectations of your patients or their families influence you to consider DCD? (patients if wishes for or against organ donation are known)
    i. If yes, how?
    ii. If no, why not?
- Do other team members influence your consideration of DCD? (prompts: decision to have a patient become a DCD donor, management of a DCD case) (co-workers / team lead / department / overall workplace)
    i. If yes, who and how?

Emotion

- What feelings do you experience when you think about DCD?
    i. The concept itself
    ii. When a patient is a candidate, when they are not
    iii. Whether a donation successfully proceeded

Goals

- On a scale of 1-10, with 10 being the most important, how important do you think it is for you to consider DCD (when appropriate)?
    i. (If not 10), what is a higher priority?

Reinforcement

- In the past, are there any personal or external incentives that you have experienced to be effective to help you use DCD? (prompt: yourself, or others)
    i. Any deterrents to using DCD?
- Does payment or non-payment to the hospital or physicians affect your use of DCD?

Behavioural regulation

- If you wanted to implement changes in your own practice to increase the use of DCD, what would be some ways to do this? (prompts: individual/team setting/practice setting)

Optimism

- What are your thoughts about the future of DCD in your province and Canada?

Thank you for taking the time to participate in our study.
